# Current Understanding of the Role of Autophagy in the Treatment of Myeloid Leukemia

**DOI:** 10.3390/ijms252212219

**Published:** 2024-11-14

**Authors:** Yasushi Kubota, Shinya Kimura

**Affiliations:** 1 Department of Clinical Laboratory Medicine, Saga-Ken Medical Centre Koseikan, Saga 840-8571, Japan; 2 Division of Hematology, Respiratory Medicine and Oncology, Department of Internal Medicine, Faculty of Medicine, Saga University, Saga 849-8501, Japan; shkimu@cc.saga-u.ac.jp

**Keywords:** acute myeloid leukemia, chronic myeloid leukemia, cyclodextrin, hydroxypropyl-β-cyclodextrin, folic acid, folate receptor, mitophagy, clinical trial

## Abstract

The most important issues in acute myeloid leukemia are preventing relapse and treating relapse. Although the remission rate has improved to approximately 80%, the 5-year survival rate is only around 30%. The main reasons for this are the high relapse rate and the limited treatment options. In chronic myeloid leukemia patients, when a deep molecular response is achieved for a certain period of time through tyrosine kinase inhibitor treatment, about half of them will reach treatment-free remission, but relapse is still a problem. Therefore, potential therapeutic targets for myeloid leukemias are eagerly awaited. Autophagy suppresses the development of cancer by maintaining cellular homeostasis; however, it also promotes cancer progression by helping cancer cells survive under various metabolic stresses. In addition, autophagy is promoted or suppressed in cancer cells by various genetic mutations. Therefore, the development of therapies that target autophagy is also being actively researched in the field of leukemia. In this review, studies of the role of autophagy in hematopoiesis, leukemogenesis, and myeloid leukemias are presented, and the impact of autophagy regulation on leukemia treatment and the clinical trials of autophagy-related drugs to date is discussed.

## 1. Introduction

Autophagy is a pathway that transports proteins and cell organelles in the cytoplasm to lysosomes for degradation [1]. Autophagy is induced when cells are starved and provides amino acids and protects cells by degrading intracellular components. Autophagy is also known to be activated when cells are exposed to environmental stresses such as hypoxia and genotoxic stress, which inhibits apoptosis. In addition, it is now clear that the constitutive activity of autophagy is sustained even under non-stressed conditions, maintaining intracellular homeostasis by degrading abnormal proteins and excess intracellular organelles. Autophagy is thought to assist cancer cells not simply through amino acid production but also through broader cellular remodeling, intracellular quality control, and inhibition of cell death, cellular senescence, and cancer immune responses [2,3,4].

Since autophagy is involved in drug resistance and survival and proliferation in the tumor environment in various solid tumors and hematological malignancies, autophagy inhibition is a promising cancer treatment concept [4]. However, there have also been reports of agents that induce autophagy in tumor cells, leading to autophagic cell death.

Cancer stem cells are located in hypoxic regions within tumors and exhibit glycolysis-dependent metabolism [5], but autophagy is activated in hypoxic conditions, and mitochondria are degraded by autophagy [6,7], so the importance of autophagy in cancer stem cells has also been pointed out. In this review, recent advances in the role of autophagy in the treatment of acute myeloid leukemia (AML) and chronic myeloid leukemia (CML) are discussed from the perspective of both autophagy activation and inhibition.

## 2. Mechanism of Autophagy

### 2.1. Autophagy Pathway

**Figure 1:**
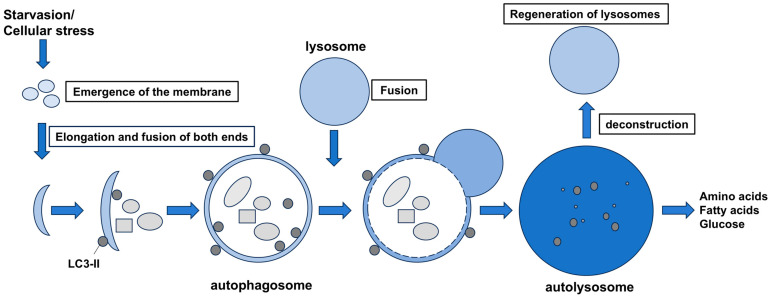
Autophagy pathway. The membrane that forms at the contact site between the mitochondria and endoplasmic reticulum extends to surround the degradation products at both ends and closes (autophagosome). After that, it fuses with the lysosome to become an autolysosome, and the internal contents are broken down by digestive enzymes. Lysosomes are regenerated from the autolysosome.

Autophagy proceeds as follows. First, a flat membrane structure called phagophore appears in the cytoplasm, which elongates at both ends to enclose the organelles and proteins to be degraded, and finally the ends fuse with each other (autophagosomes). Autophagosomes have a double membrane and then fuse with lysosomes to become autolysosomes. Inside the autolysosome, a group of lysosome-derived hydrolytic enzymes degrades the contents of the internal envelope, producing degradation products such as amino acids, fatty acids, and glucose, which are reused as materials and energy for protein synthesis necessary for cells (Figure 1).

**Figure 2:**
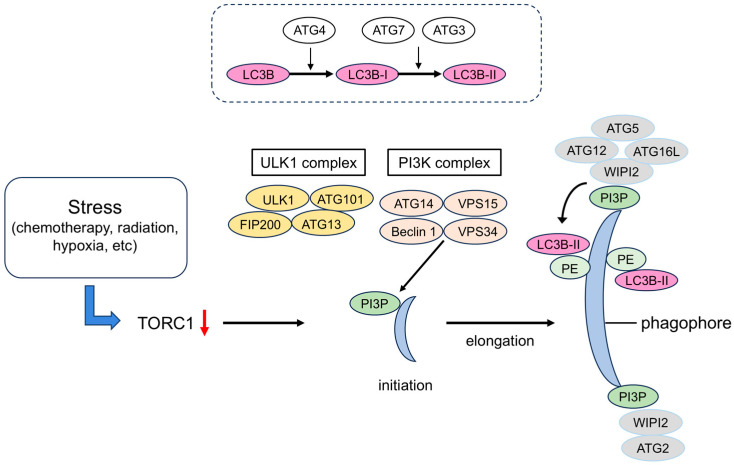
Formation of autophagosomes. The ULK1 complex, which is involved in the initiation of autophagy, is inhibited by the mTORC1 kinase complex, so autophagy is induced when TORC1 is inactivated by factors such as nutrient starvation. When the ULK1 complex migrates to a subdomain of the endoplasmic reticulum (ER), the PI3K complex I is recruited, and the production of PI3P production increases. The PI3P-binding protein WIPI binds to it and accumulates at the site of autophagosome formation together with its partner ATG2. ATG2 anchors the ER and the phagophore and transports lipids. The ATG12 system is a system in which ATG12 and ATG5 are covalently bound to each other via a ubiquitin-like binding reaction. The ATG12–ATG5 complex forms a ternary complex with ATG16L and localizes to the phagophore, where it determines the location of amide bond formation between ATG8 family proteins (LC3B) and PE. LC3B-PE localizes to the inner and outer membranes of the phagophore and autophagosome, and it functions in membrane elongation and closure. TORC1, target of rapamycin complex 1; ULK1, Unc51-like kinase 1; PI3K, phosphatidylinositol-3 kinase; PI3P, phosphatidylinositol-3-phosphate; WIPI, WD repeat domain phosphoinositide-interacting; ATG, autophagy-related protein; LC3B, light chain 3B; FIP200, focal adhesion kinase interacting protein; VPS34, vacuolar protein sorting 34.

The mechanism of autophagy has been studied in yeast. A number of autophagy-related (ATG) proteins have been isolated and purified [8], and a network of ATG is involved in autophagy [9]. The proteins involved in the formation of autophagosomes are called “core ATG proteins” and are divided into six functional units: (1) Unc-51 like autophagy activating kinase 1 (ULK1) protein kinase complex, (2) ATG9 vesicle, (3) phosphatidylinositol 3-kinase complex I (PI3K complex I), (4) ATG2–WD-repeat protein interacting with phosphoinositides (WIPI) complex, (5) ATG12–ATG5–ATG16L complex, and (6) ATG8 family protein–phosphatidylethanolamine (PE) binding reaction system. When autophagy is induced, these functional units accumulate at the site of autophagosome formation to construct a pre-autophagosomal structure (PAS). The formation of the phagophore occurs through the sequential action of these six functional units [10,11] (Figure 2).

In particular, ATG12–ATG5 binding is important for the formation of autophagosomes, and autophagosomes are not formed when ATG5 is deficient [12]. In addition, the presence of light chain 3 (LC3) (mammalian homologue of ATG8) translocation to the membrane (converted from LC3-I to LC3-II when translocated to the autophagosome) is essential for autophagy and serves as a biochemical marker. The curved phagophore continues to elongate due to the action of ATG7 and ATG10, which form the ATG5–ATG12–ATG16L1 complex, creating a ubiquitin-like activating enzyme. The ATG7–ATG3 complex is involved in the lipidation of LC3 with the help of ATG4, and as a result, LC3-II is formed on the surface of the autophagosome [13].

### 2.2. Autophagy Signaling in Cancer

The link between autophagy and cancer first came to attention in 1999, when Liang, et al. proposed that *BECN1*, a mammalian homolog of *ATG6/VPS30*, an essential gene for yeast autophagy, is a tumor suppressor gene [14]. In fact, it has been reported that heterozygous *Becn1*-deficient mice are susceptible to liver cancer, lung cancer and lymphoma [15,16], and that a single allele of *BECN1* is frequently deleted in breast cancer, ovarian cancer and prostate cancer [17]. Subsequently, it was also discovered that genetically modified mice specialized in autophagy (systemic *Atg5* mosaic-deficient mice and liver-specific *Atg7*-deficient mice) also formed liver tumors, and tumor suppression was firmly established as one of the physiological functions of autophagy [18,19].

Under stress, cancer cells activate autophagy. Stress includes an increase in the number of cancer cells, chemotherapy, radiation, hypoxia, and the accumulation of toxic waste, which cause the inactivation of mammalian target of rapamycin (mTOR) and the activation of AMP-activated protein kinase (AMPK) to induce autophagy. The development of phagophores or crescent-shaped double-membrane structures is promoted, and the Unc-51-like kinase (ULK) complex is mainly involved in this step. The yeast homolog of ATG1, which is composed of the main signaling factors of this initiation complex, is ULK1, and there are also several ULK isoforms. ULK2 can sometimes compensate for the role of ULK1, but the roles of the other isoforms are largely unknown. ULK1 is highly expressed in colon cancer, breast cancer, cervical cancer, and AML, but its exact role in cancer has not yet been fully elucidated [20,21,22,23]. Recently, in a study of non-small cell lung cancer (NSCLC) with a mutation in liver kinase B1 (LKB1), it was reported that inhibiting ULK1 interferes with the initiation stage of autophagy, which has a synergistic effect with the inhibition of programmed cell death protein-1 (PD-1) antibody and that it leads to an increase in the effector T-cell population and tumor regression [24]. It is known that BRAF-positive thyroid tumors survive by inducing cell-protective autophagy via the LKB1–AMPK–ULK1 pathway. In addition to BRAF inhibition, autophagy inhibition induces apoptosis [25]. The ULK complex is composed of ULK1, ATG13, ATG101, and the 200 kDa focal adhesion kinase-interacting protein (FIP200), which is activated when mTORC1 is inhibited or the AMPK pathway is activated [26]. The pre-autophagosome protein ATG14 is recruited to the contact site between the endoplasmic reticulum and mitochondria to form autophagosomes [27]. ATG14 plays an important role in regulating oxidative stress, and the lipophagy induced by ATG14 leads to the accumulation of reactive oxygen species (ROS), which regulates ER-mediated mitochondrial apoptosis in HeLa cells [28].

## 3. Autophagy and Hematopoiesis

### 3.1. Autophagy and Normal Hematopoietic Stem Cells

Autophagy is a mechanism present in cells throughout the body that has been found to play an important role in the maintenance of normal hematopoietic stem cells. In experiments using mice in which *Atg7* or *FIP200*, genes essential for autophagy, were conditionally knocked out in blood cells, respectively, it was shown that the knockout of these genes in adult and fetal hematopoietic stem cells (HSCs) caused an increase in mitochondria and accumulation of ROS, leading to a marked decrease in the number of adult HSCs or fetal HSCs [29,30]. A similar phenotype has been reported in *Atg5*-deficient mice [31,32]. It has been reported that autophagy also regulates the cell cycle in a nutrient-dependent manner [33]. Since mouse HSCs maintain low mitochondrial activity, autophagy may contribute to HSC maintenance through mitochondrial degradation [34]. Furthermore, mouse HSCs suppress cell death by activating autophagy via FoxO3a under conditions of metabolic stress, and old HSCs have high autophagic activity and increased dependency on autophagy even at steady state [35]. Granulocyte-colony stimulating factor (G-CSF) activates autophagy in HSCs and neutrophils, and G-CSF-induced mobilization of HSCs to the periphery is impaired by the absence of autophagy [36]. In human HSCs, autophagic activity has been shown to be higher than in differentiated cells, and analyses using immunodeficient mice have confirmed that the knockdown of *ATG5* or *ATG7* reduces hematopoietic reconstitution ability [37,38]. Taken together, autophagy is essential for the development and maintenance of normal HSCs [39], and the dependence on autophagy increases with stress, particularly with aging and exposure to environments outside the bone marrow.

### 3.2. Autophagy and Leukemogenesis

Analyses using leukemia mouse models have been conducted to elucidate the role of autophagy in the initiation and progression of leukemia. In a CML mouse model in which *BCR::ABL*-transfected mouse bone marrow cells were transplanted into immunocompromised mice, the knockout of *Atg3* after engraftment significantly delayed the initiation of CML [40]. This indicates that autophagy is essential for the pathogenesis of CML. In the AML model of transplantation of *MLL::AF9*-transfected mouse bone marrow cells, the knockout of *Atg5* after transplantation also prolonged mouse survival [41]. However, it has been reported that the heterozygous knockout of *Atg5* shortens survival in a mouse leukemia model caused by MLL::ENL [37]. Therefore, further studies of the effects of different degrees of autophagy activity on the progression of myeloid leukemia are warranted. Pre-mRNA splicing factor U2AF35-transformed cells showed an aberrant processing of *ATG7* pre-mRNA, resulting in a longer 3′ UTR and reduced ATG7 expression level. Decreased ATG7 led to an autophagy defect, mitochondrial dysfunction, genomic instability, and the transformation of MDS to AML [42]. In the mixed lineage leukemia–eleven nineteen leukemia-induced AML model, the role of autophagy in leukemia-initiating cells (LICs) was analyzed by the loss of *Atg5* or *Atg7*. Results showed a decrease in LICs and prolonged survival of leukemic mice. Accompanying this, increased mitochondrial accumulation and the production of reactive oxygen species in the LIC fraction, as well as increased cell death, were observed [43].

The influence of autophagy in differentiated leukemia cells, as well as in stem cell fractions, has been investigated. In acute promyelocytic leukemia (APL) with t(15;17) translocation, autophagy-linked FYVE domain-containing protein (ALFY/WDFY3) is a PI3P-interacting autophagy scaffold protein [44], and its mRNA increased in APL cells during differentiation induction therapy with all-trans retinoic acid (ATRA). ALFY depletion resulted in impaired ATRA-induced granulocytic differentiation of APL and AML cells [45]. The expression of the ATG gene family was low in primary AML cells compared with healthy granulocytes, and the inhibition of ATG3, ATG4D, and ATG5 impaired AML cell differentiation with ATRA [46].

In comparison with the CD34-positive fraction, which is considered to contain a large number of leukemic stem cells (LSCs), CD34-positive cells derived from CML patients had a higher expression of the autophagy-related gene *ATG4B* than normal bone marrow CD34-positive cells, and *ATG4B* knockdown reduced cell proliferative capacity and colony-forming ability [47].

## 4. Autophagy and AML Subtypes

### 4.1. Autophagy in NPM1-Mutated AML

AML with mutated nucleophosmin member 1 (*NPM1)* is more common in de novo AML with normal karyotype and generally has a better prognosis. Exon 12 mutations, which alter the subcellular localization of the NPM1 protein, are common. Cases of acute monocytic leukemia and acute myelomonocytic leukemia are more frequently associated with *NPM1* mutations and are associated with these morphological features. In *NPM1*-mutated AML, mRNA levels of autophagy-associated genes were elevated [48]. A feedback loop has been proposed in which the activation of autophagy leads to the proteolysis of mutant NPM1 and hexamethylene bisacetamide-inducible protein 1 (HEXIM1) and the subsequent activation of Brd4, which in turn contributes to the continued activation of autophagy [49]. *NPM1* mutation type A (NPM1-mA) interacts and mediates promyelocytic leukemia gene (PML) delocalization to the cytoplasm. This enhances PML stabilization and consequently promotes autophagy via AKT signaling, leading to leukemic cell survival [50].

Pyruvate kinase isoenzyme M2 (PKM2) is highly expressed in *NPM1*-mutated AML. PKM2 mediated the activation of autophagy and increased the phosphorylation of Beclin-1. Importantly, PKM2 contributes to leukemic cell survival via the activation of autophagy, and high PKM2 expression is associated with poor clinical outcomes in *NPM1*-mutant AML patients [51]. ULK1, a core protein that mediates autophagosome formation, is also highly expressed in *NPM1*-mutant AML. *NPM1*-mA maintains ULK1 protein stability by promoting TRAF6-dependent ubiquitination, which is essential for autophagy activation and leukemic cell survival [52].

The involvement of long noncoding RNAs (lncRNAs) in the pathogenesis and prognosis of AML has also been reported [53,54]. In *NPM1*-mutated AML, Hox antisense intergenic RNA myeloid 1 (HOTAIRM1) was highly expressed and promoted leukemia cell autophagy by regulating early growth response 1 (EGR1) and unc-51-like autophagy activating kinase 3 (ULK3) [55]. Tumor protein p53 inducible nuclear protein 2 (TP53INP2) acts as a transcription factor in the nucleus, but it translocates to the cytoplasm under nutrient starvation and is involved in autophagy regulation [56]. In *NPM1*-mutated AML, cytoplasmic TP53INP2 promoted the interaction between LC3 and ATG7, which in turn enhanced autophagy. This activation of autophagy led to the maintenance of leukemia cell survival [57]. Gamma aminobutyric acid A receptor-associated protein (GABARAP), a member of the human ATG8 family, also participated in autophagy activation of the NPM1c variant [58].

### 4.2. Autophagy and FLT3-Mutated AML

The FMS-like tyrosine kinase 3 (FLT3) molecule, which is involved in cell differentiation and proliferation, is expressed in most AML cases, and the *FLT3*-ITD mutation, which is found in approximately 30% of AML cases, is one of the factors associated with a poor prognosis [59]. Allogeneic hematopoietic stem cell transplantation is recommended during the first remission period for *FLT3*-ITD-positive AML, but the high rate of relapse after transplantation is a problem compared with cases with *FLT3*-ITD-negative AML [60]. Therefore, the emergence of other promising drugs for post-transplant treatment targeting the FLT3 signal is anticipated.

The inhibition of FLT3-ITD induced a pro-cell death lipid, ceramide-dependent mitophagy. The mechanism is as follows: ceramide accumulates on the outer mitochondrial membrane, and this directly binds to autophagy-inducing LC3 for recruiting autophagosomes [61]. Ret proto-oncogene (RET) protein is highly expressed in AML cells and suppresses autophagy via mTORC1 signaling. Pharmacological and genetic *RET* inhibition led to the autophagic degradation of FLT3 and depletion of *FLT3*-mutated AML cells, suggesting that disruption of RET signaling is a potential therapeutic strategy for *FLT3*-mutated AML [62].

AKT–mTORC1–ULK1-dependent autophagy was identified as a primary resistance mechanism to FLT3 kinase inhibitors (FLT3is) using translatome proteomics [63]. Treatment with an FLT3i combined with an autophagy inhibitor, ROC-325 and Lys05 [64,65], showed a synergistic antileukemic effect in the *FLT*-ITD-positive mouse model and primary *FLT3*-ITD-positive AML cells [63]. Zhang et al. found a positive association between the increase in phospho-Bruton tyrosine kinase (BTK) and autophagy in FLT3 inhibitor-resistant AML cells. GC-806 (luxeptinib), a small molecule kinase inhibitor that blocks FLT3, BTK, and aurora kinases, showed efficient antileukemia activity in FLT3i-resistant leukemias in vitro and in vivo [66]. A phase 1 clinical trial of GC-806 in relapsed/refractory AML is ongoing [67].

### 4.3. Autophagy and TP53 in AML

*TP53* mutations are abnormalities that directly inactivate p53. *TP53* mutations are generally found in approximately half of solid tumors, and *TP53* mutations are particularly frequent in cancers with a poor prognosis [16,68]. This is also true for hematological malignancies, in which *TP53* mutations are as frequent as 70% in MDS/AML with a complex aberrant karyotype with a poor prognosis [69].

The inhibition of autophagy by hydroxychloroquine (HCQ) and ATG5 or ATG 7 was effective in leading wild-type p53 AML cells to apoptosis but not the *p53*-mutated AML cells. Furthermore, HCQ induced BCL2-associated X (BAX) and p53-upregulated modulator of apoptosis (PUMA)-dependent apoptotic responses in AML cells with normal p53 but not in the p53 mutant [70]. In many cancers, p53-R248Q is the most frequent mutant [71,72,73]. Strategies to eliminate mutant p53 proteins are attractive for treating mutant p53 tumors and potentially improving the prognosis of cancer patients. Heat shock protein 90 (Hsp90) inhibition by 17-allylamino-17-demethoxygeldanamycin (17-AAG) degraded R248Q by stimulating macroautophagy under normal conditions; in contrast, under conditions of metabolic stress, 17-AAG promoted R248Q binding to Hsc70 and activated chaperone-mediated-autophagy (CMA) [74]. Casein kinase 1α (CSK1α), encoded by CSKN1A1 and negative regulator of Wnt/β-catenin and p53 signaling pathways [75], was highly expressed in newly diagnosed AML, and the survival analysis of AML patients using a TCGA-LAML database showed that CSKN1A1 mRNA expression was correlated with the survival of AML patients. The inhibition of CK1α using D4476 or shRNA induced apoptosis and autophagy in AML cells [76]. Fructose-bisphosphatase 1 (FBP1) is a glyconeogenic enzyme that is essential for carbohydrate metabolism. The MV4-11 cell line overexpressing FBP1 (namely FBP1-MV4-11) increased P53 protein and was shown to be more prone to apoptosis than MV4-11 blast cells. Furthermore, FBP1-MV4-11 cells had impaired mitochondrial homeostasis, suggesting that FBP activates P53 and mitophagy in AML blasts [77]. A recent study showed that X-chromosome-linked inhibitor of apoptosis protein (XIAP) is also involved in the regulation of autophagy in AML. XIAP inhibition decreased p53 and upregulated autophagy by modulating AMPK/mTOR signaling [78]. Tropomodulin 1 (TMOD1) is a diagnostic marker for several tumors [79,80,81]. Xia et al. found a high expression of TMOD1 in AML cells. The silencing of TMOD1 enhanced Karyopherin subunit alpha 2 (KPNA2) stability and increased the nuclear transfer of p53, triggering autophagy and inhibiting AML cell growth [82].

## 5. Autophagy Modulation in Acute Myeloid Leukemia

In the field of AML, there are also many reports of drugs that induce or inhibit autophagy. Promising therapeutic agents to target AML cells via autophagy have been found and investigated.

### 5.1. Activation of Autophagy

Vitamin D3 induces autophagy, as well as differentiation, in AML cells [83,84,85,86]. As a mechanism inducing autophagy, it was found that vitamin D inhibits miR-17-5p-induced Beclin-1 overexpression [87]. In APL, it has been shown that treatment with ATRA or arsenic acid (ATO) induces autophagy, which is essential for the degradation of PML::RARα [88]. The inhibition of autophagy with chloroquine and 3-methyladenine (3-MA) or shRNA knockdown attenuated ATRA-induced cell differentiation, suggesting that autophagy is an important process in ATRA-induced APL cell differentiation [89]. Dendrogenin A (DDA) is a cholesterol metabolite with tumor-suppressor activity that induces lethal autophagy by activating liver-X-receptor β (LXRβ) and inhibits the cholestrogenic function of 3β-hydroxysterol-Δ8,7-isomerase (D8D7I) [90]. The combination of DDA and cytarabine (Ara-C) or idarubicin synergistically suppressed the growth of AML cells in vitro and in vivo [91,92].

A synergistic effect between the anti-diabetic drug metformin and the FLT3 inhibitors has been reported for *FLT3*-ITD-positive AML. The underlying mechanism is thought to be the induction of autophagy by inhibiting the mTOR pathway and the suppression of Polo-like kinase 1 (PLK1) expression [93,94]. AC-73 is an inhibitor of CD147, which is a type I transmembrane glycoprotein that is highly expressed in various types of cancer [95]. Spinello et al. found that AC-73 inhibits AML cell growth by blocking ERK/STAT3 signaling and activates autophagy. In addition, AC-73 in combination with Ara-C or ATO enhanced the antileukemia effects of each drug [96]. Quercetin, a member of the flavonoid family, induced apoptosis and autophagy in AML cells through the AMPK/mTOR signaling pathway [97]. Resveratrol (3,5,4′-trihydroxy-trans-stilbene) is a naturally occurring polyphenol with anti-tumor activities [98]. Resveratrol induced both apoptosis and autophagy in HL-60 cells by regulating the PI3K/AKT and LKB1/AMPK/mTOR signaling pathways [99,100].

Neratinib is a tyrosine kinase inhibitor approved for the treatment of human epidermal growth factor receptor 2-positive breast cancer [101]. Ma et al. investigated the effect of neratinib in AML cell line HL-60, and they showed that neratinib suppresses the proliferation of AML cells through autophagy-dependent ferroptosis [102]. Ferroptosis is a type of “controlled cell death” that is caused by the accumulation of iron-dependent lipid peroxides [103]. It has also recently been attracting attention as a new target for cancer treatment [104]. 4-Amino-2-trifluoromethyl-phenyl retinate (ATPR) is a derivative of ATRA that was developed to be more effective than ATRA in AML [105]. It has also been reported that autophagy and ferroptosis are involved in the differentiation of leukemia cells by ATPR [106,107]. Dihydroartemisinin (DHA), an FDA-approved antimalarial drug, inhibited the proliferation of AML cells by inducing ferroptosis. It is thought that the mechanism is as follows: DHA activates AMPK phosphorylation, downregulates the mTOR/p70S6k pathway, and induces autophagy, resulting in ferroptosis [108].

More recently, it has been reported that NCOA4-mediated ferritinophagy [109] is important for maintaining AML LSCs. Comparative proteomic analysis using a patient-derived xenograft (PDX) model of AML showed that iron metabolism is an important distinguishing factor for quiescent AML LSCs, and ferritinophagy maintains the iron bioavailability of these cells [110]. The small-molecule inhibitor compound 9a, which inhibits the interaction between NCOA4 and ferritin [111], selectively targeted AML LSCs.

Folate-appended hydroxypropyl-β-cyclodextrin (FA-HP-β-CyD) induced autophagic cell death not only in CML but also in AML. It showed a synergistic effect when used in combination with cytarabine or venetoclax, and it also extended the survival period of AML mouse models [112].

### 5.2. Inhibition of Autophagy

Hydroxychloroquine (HCQ), a chloroquine derivative, has been widely used to block late-stage autophagy. HCQ induced apoptosis in AML cells by inhibiting autophagy, and its growth inhibitory effect was more potent in Ara-C-resistant U937 cells than in Ara-C-sensitive cells [113]. The effect of HCQ in primary human CD34-positive AML cells was examined. The sensitivity to HCQ was higher in AML CD34-positive cells than in normal bone marrow CD34-positive cells, and when the cells were divided into those with high and low ROS levels, autophagy activity was higher in the ROS low group, and HCQ was more effective [70]. However, in Kmt2a/Mll/Mllt3/Af9 AML, HCQ was ineffective in vivo because of vesicular exocytosis [114]. Since AML is a heterogeneous disease, there are also reports that the effects of HCQ are not uniform [115]. In addition, autophagy inhibitors containing CQ were unable to re-sensitize Ara-C-resistant AML cells [116].

Bafilomycin A1 (Baf A1) is a specific inhibitor of vacuolar H+-ATPase and is also used for inhibiting late-stage autophagy [117]. Of three autophagy inhibitors including 3-methyladenine (3-MA), CQ, and Baf A1, Baf A1 treatment showed the most significant reduction in colony numbers in the AML CFU assay, whereas it had almost no effect on cord blood-derived normal hematopoietic progenitors. Furthermore, the combination of Baf A1 and Ara-C significantly reduced AML tumor burden in an in vivo AML mouse model compared with Ara-C alone [118].

SA405 is a vacuolar protein sorting 34 (Vps34) inhibitor that prevents autophagy [119]. Treatment with SAR405 inhibited autophagy and suppressed the proliferation of *FLT3*-ITD-positive AML cell lines, MOLM-14 and MV4-11 cells, in vitro [120]. The combination of SAR405 and mobilizing agents strongly reduced relapse in the *FLT3*-ITD AML xenograft model in vivo [121]. VSP34-IN1 is another specific VPS34 inhibitor [122], and it inhibits both basal autophagy and L-asparaginase-induced autophagy in AML cells [123]. VPS34-IN1 inhibited mTORC1 signaling and STAT5 phosphorylation in *FLT3*-ITD AML [123].

p62/SQSTM1 is a receptor for ubiquitin-mediated selective autophagy [124]. XRK3F2 is a ZZ-domain of a p62 (p62-ZZ) inhibitor identified using 3D homology modeling and molecular docking studies [125]. Li et al. used XRK3F2 to evaluate the mechanisms of mitophagy in the survival of AML LSCs, and they showed that XRK3F2 selectively attenuated leukemia-initiating ability, but it preserved normal HSCs by blocking mitophagy [126]. TAK-165, a HER2 inhibitor, was selected from among 12,640 compounds through small-molecule screening to identify a compound that shows a synergistic effect when used in combination with AC220 (quizartinib). TAK-65 inhibited autophagy in AML cells; interestingly, its combination with AC220 showed the activation of chaperone-mediated autophagy in a HER2-independent manner [127].

An autophagosome inhibitor, MRT-68921, in combination with ATRA induced irreversible differentiation in AML cells by activating the DNA sensor, which was absent in melanoma 2 (AIM2). In addition, MRT-68921 + quizartinib synergistically induced irreversible differentiation in AML cells carrying an *FLT3*-ITD [128].

## 6. Autophagy and Chronic Myeloid Leukemia

The therapeutic outcome of CML improved dramatically with the advent of tyrosine kinase inhibitors (TKIs), and long-term survival has become possible [129]. Second- and third-generation TKIs have been developed for cases in which point mutations within the ABL kinase domain have emerged and resulted in TKI resistance [130]. Recently, several groups have shown that 40% of patients who maintain complete molecular response (CMR) for a certain period of time after the initiation of TKIs can remain in CMR for a long period of time after TKI discontinuation, and that functional cure, termed treatment-free remission (TFR), may be achieved [131,132,133,134,135,136]. However, this simultaneously indicates that about half of the remaining cases relapse after TKI discontinuation. In all of these relapsed cases, sensitivity to repeat TKI therapy was maintained, suggesting the existence of CML stem cells that are independent of BCR::ABL signaling and viable within the bone marrow microenvironment even under conditions of long-term remission [137,138]. CML stem cells, like normal HSCs, are thought to be able to survive for long periods of time while maintaining an undifferentiated and quiescent state [139]. Targeting and eradicating these CML stem cells that form minimal residual disease (MRD) may lead to a complete cure of CML.

Autophagy is involved in chemoresistance and cell survival and growth in the tumor environment in various solid tumors and hematopoietic malignancies [4]. Therefore, autophagy inhibition has attracted attention as a promising cancer treatment concept. CD34-positive cells derived from CML patients have a higher expression of ATG4B, an autophagy-related gene, than normal bone marrow CD34-positive cells, and the suppression of autophagy by the knockdown of *ATG4B* resulted in decreased cell proliferative capacity and colony-forming ability [140]. Metabolomic analysis after the knockdown of *ATG7* in CML cell lines showed that the inhibition of autophagy increases mitochondrial activity and oxidative phosphorylation (OXPHOS), which is followed by differentiation into the erythroid lineage [141]. Reports that imatinib induces autophagy also underscore the link between CML and autophagy [142,143]. Other TKIs, such as bafetinib (INNO-406) and dasatinib, have also been reported to induce autophagy [144,145]. It has also been reported that the inhibition of autophagy enhances the cell-killing effect of daunorubicin on CML cells in vitro [146].

Chloroquine (CQ) and its less toxic derivative, HCQ, are used as autophagy inhibitors, and they are the only two autophagy inhibitors that are approved by the Food and Drug Administration (FDA) [147]. Bellodi et al. showed that the combination of TKIs with CQ led to the death of TKI-resistant CML stem cells [142]. This result led to the CHOICES study, which is a phase II clinical trial to evaluate the efficacy of the combination of HCQ and imatinib, but even using the maximum tolerated doses of HCQ, it was difficult to obtain consistent autophagy inhibition, and the trial failed to demonstrate clear efficacy [148]. A more potent second-generation autophagy inhibitor, Lys05 [65], reduced the number of primary CML stem cells, and *in vivo* models showed that it could eradicate CML stem cells in combination with TKIs [149].

Mammalian target of rapamycin (mTOR), discovered as a target molecule of rapamycin, is a phosphatase that regulates various cellular physiological responses and plays important roles in protein translation, cytoskeletal regulation, and autophagy control by sensing nutrient and energy status and stress [150]. Activation of the mTOR pathway induced by eutrophic conditions and growth factors is closely related to abnormal glycolipid metabolism and carcinogenesis. Mitchell et al. generated a ponatinib-resistant CML cell line (KCL22^pon-res^) and performed RNA-sequencing (RNA-seq) and gene ontogeny (GO) enrichment analysis to detect alternative drug targets in BCR::ABL-independent TKI resistance [151]. The activation of mTOR was highlighted as a potential target for TKL resistance. Of the catalytic mTOR inhibitors, the growth inhibitory effect of NVP-BEZ235, which inhibits activities of PI3K and mTORC1/mTORC2 [152,153], on TKI-resistant CML cells was investigated in detail. NVP-BEZ235 induced autophagy, and its combination with HCQ significantly prolonged the survival of CML-xenografted mice. A phase I trial of NVP-BEZ235 was conducted based on these results, but it did not show benefit in relapsed or refractory myeloid leukemias [154].

Autophagy has also been implicated in the effects of histone deacetylase (HDAC) inhibitors. Analysis of human CML cell lines and patient samples showed that CQ selectively increased the sensitivity of leukemic cells to HDAC inhibitors [155]. More recently, chidamide has been reported to be effective in T315I-resistant CML cells through the Akt-autophagy pathway [156]. We recently found that folate-appended hydroxypropyl-β-cyclodextrin (FA-HP-β-CyD) induces autophagic cell death in CML cells [157]. Unmodified HP-β-CyD also exhibits antileukemic activity, but it does not enter cells and is also nonselective for tumor cells [158]. In contrast, FA-HP-β-CyD appears to be taken up into cells via the folate receptor (FR) and induces autophagy. In contrast to HP-β-CyD, FA-HP-β-CyD shows no cytotoxic effect on the A549 lung cancer cell line, which does not express FR, and is effective against myeloid leukemia cells that strongly express FR [157].

## 7. Clinical Trials Using Autophagy Modulators

At this time, no compounds that specifically inhibit or promote autophagy have been found, and therapies targeting autophagy are limited. Already approved drugs that can induce autophagy are being tested in clinical trials as an addition to conventional therapy (Table 1).

The proteasome inhibitor bortezomib was used in several trials. Howard et al. reported the result of a phase I study using bortezomib with weekly idarubicin in newly diagnosed elderly AML or any relapsed AML patients. The CR rate was 20%, and the combination of bortezomib and idarubicin was well tolerated [159]. Attar et al. added bortezomib to standard 3 + 7 daunorubicin and cytarabine induction chemotherapy for previously untreated AML. This study reported that the CR rate was 65% with an overall response rate (ORR: CR+CRp) of 69% [160]. In patients with APL relapsing after upfront ATO therapy, a phase 2 study using bortezomib and ATO was performed. Of the 21 evaluable patients, 19 (90.5%) achieved molecular remission after induction therapy. Although one patient required the discontinuation of treatment for grade 3 neuropathy, bortezomib could be continued in addition to the salvage regimen with ATO and ATRA [161]. For pediatric patients with relapsed or refractory AML, bortezomib was added to salvage regimens, and its tolerability and efficacy were evaluated. The trial was closed because it did not reach the predetermined efficacy thresholds, although the CR rates were approximately 50% [162].

The combination of mTOR inhibitors and conventional chemotherapeutic drugs has also been evaluated for efficacy in several clinical trials. Sirolimus, an mTORC1 inhibitor, was administered along with the MEC regimen (mitoxantrone, etoposide, and cytarabine) to patients with relapsed/refractory AML with an ORR of 47% [163]. The CR rate (CR+CRp 35%) was higher than historical data of MEC in similar populations (CR 21%) [164]. A phase II study of temsirolimus, a water-soluble ester analog of sirolimus, in combination with low-dose clofarabine was conducted in elderly patients with relapsed/refractory AML. The ORR rate was 21%. In this study, an in vivo inhibition of mTOR signaling was evaluated by measuring S6 ribosomal protein (S6RP) phosphorylation in bone marrow, and a high inhibition rate (>50%) correlated with clinical responses (CR 75% vs. 0% without inhibition) [165]. The combination of RAD001 (everolimus) and conventional induction chemotherapy (3 + 7 regimen) achieved CR in 65% of patients with relapsed AML aged 65 years or younger [166]. A phase Ib trial of everolimus with low-dose cytarabine (LDAC) for unfit elderly patients with AML showed an ORR of 25% [167].

Devimistat (CPI-613) is an analog of lipoic acid that inhibits the TCA cycle [168,169]. The inhibition of the TCA cycle by devimistat induces autophagy-dependent mitochondrial turnover, and AML cells treated with devimistat were more sensitive to CQ in vitro [170]. Clinical trials were conducted involving patients with relapsed or refractory AML using a combination of devimistat with high-dose cytarabine and mitoxantrone (HAM) salvage chemotherapy. A pooled analysis of phase I and II trials using devimistat in combination with HAM therapy showed a CR rate of 52% and a median OS of 10.4 months [170,171]. However, the results of the phase III ARMADA 2000 trial showed a CR rate of 20.6% and a median OS of 8.9 months, which did not show a significant improvement compared with the control arm [172].

Clinical studies using autophagy-inhibiting agents were also reported. Chidamide was developed as an HDAC inhibitor and has been reported to also inhibit autophagy [173]. In a clinical trial in relapsed/refractory AML, chidamide showed efficacy, particularly in those with epigenetic and transcription factor-related gene mutations except for *FLT3*-ITD [174]. A phase I study of HCQ combined with mitoxantrone and etoposide (NCT02631252) was planned in patients with relapsed AML; however, the study was discontinued due to low case enrollment [175]. In CML, the CHOICES (CHlorOquine and Imatinib Combination to Eliminate Stem cells) trial, a randomized phase II trial, was conducted. This study compared the safety and efficacy of HCQ and imatinib (IM) and IM alone in CML-CP patients. There was no significant difference in reducing residual disease detectable by qPCR [148]. More potent and specific autophagy inhibitors are needed to eradicate CML stem cells.

**Table 1:** Clinical trials using autophagy modulators for myeloid leukemias.

Agent	Regulation of Autophagy	Disease	Trial ID	Phase	Patients, N	Median Age (years)	Other Treatments	Clinical Response (N)	References
Bortezomib	induction	Elderly AML/relapsed AML	NCT00382954	1	20	68 (elderly) 58 (relapsed)	once weekly idarubicin	CR 20% (4/20) PR 5% (1/20)	Howard et al. 2013 [159]
		Elderly AML	NCT00742625	1/2	95	67	3 + 7 (daunorubicin + cytarabine)	CR 65% (62/95) CRp 4% (4/95) PR 2% (2/95)	Attar et al. 2013 [160]
		Relapsed/refractory AML	NCT00666588	2	46	Arm A: 10 Arm B: 6.1	Arm A: IDA + LDAC Arm B: ETP + HDAC	(Arm A) CR 29% (Arm B) CR 43%	Horton et al. 2014 [162]
		Relapsed APL	NCT01950611	2	22	26.5	ATRA + Arsenic trioxide	Molecular remission 90.5% (19/21)	Kulkarni et al. 2020 [161]
Temsirolimus	induction	Relapsed/refractory AML	NCT00775593	2	53	69	Lower-dose clofarabine	CR 8% (4/53) CRi 13% (7/53) PR 2% (1/53)	Amadori et al. 2011 [165]
RAD001 (everolimus)	induction	First relapsed AML	NCT01074086	1b	28	53.5	3 + 7 (daunorubicin + cytarabine)	CR 68% (19/28)	Park et al. 2013 [166]
		Elderly AML	NCT00636922	1b	24	74	Low-dose Ara-C	CR+CRi 25% (6/24) PR 4.2% (1/24)	Tiong et al. 2018 [167]
Sirolimus	induction	High-risk AML	NCT00780104 NCT01184898	1/2	51	60	MEC (mitoxantrone, etoposide and cytarabine)	CR 33% (17/51) CRi 2% (1/51) PR 12% (6/51)	Kasner et al. 2018 [163]
BEZ235	induction	Relapsed/refractory AML CML-BP	NCT01756118	1	24 (AML: 12) (CMLBP: 1)	61 *	none	clear response 12.5% (3/24) **	Lang et al. 2020 [154]
Devimistat	induction	Relapsed/refractory AML	NCT01768897	1	67	60	high dose cytarabine + mitoxantrone	CR 42% (26/62) CRi 8% (5/62)	Pardee et al. 2018 [171]
	induction	Relapsed/refractory AML	NCT02484391	2	48	64	high dose cytarabine + mitoxantrone	CR 34% (15/44) CRi 13% (6/44)	Anderson et al. 2022 [170]
	induction	Relapsed/refractory AML	NCT03504410	3	200	65	high dose cytarabine + mitoxantrone	CR 20.4%	Pardee et al. 2024 [172]
Chidamide	inhibition	Relapsed/refractory AML	NCT02886559	1/2	93	40	decitabine, CAG	CR 26% (24/93) CRi 20% (19/93)	Wang et al. 2020 [174]
Hydroxychloroquine	inhibition	CML-CP	NCT01227135	2	32	50	Imatinib	IM/HCQ: MMR 92% IM: MMR 80%	Horne et al. 2020 [148]

CR, complete remission; CRi, complete remission with incomplete hematopoietic recovery; CRp, complete remission with imcomplete platelet recovery; PR, partial response; ATRA, all trans retinoic acid; IDA, idarubicin; ETP, etoposide; LDAC, low-dose Ara-C; HDAC, high-dose Ara-C; IM, imatinib; HCQ, hydroxychloroquine; MMR, major molecular response; CAG, low-dose cytarabine, aclarubicin and granulocyte colony-stimulating factor; * Median age of all study participants, ** Response was seen only in ALL patients.

## 8. Conclusions

**Figure 3:**
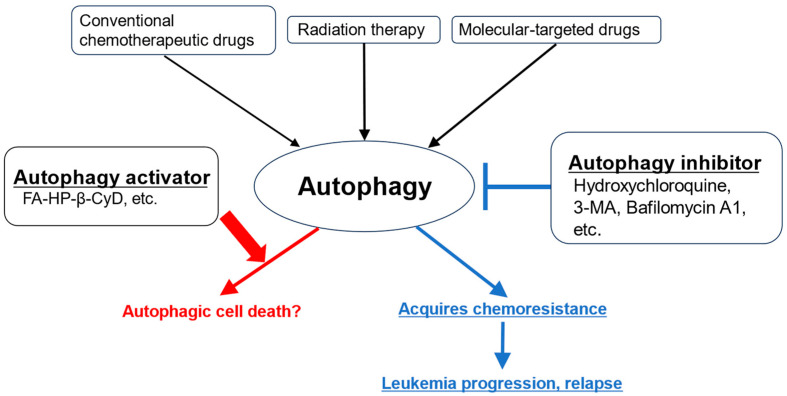
Effects of activating and inhibiting autophagy on leukemia cells. Although various anticancer drugs and radiation therapy have antitumor effects, they also activate autophagy in leukemia cells. This leads to resistance to antileukemia therapy and the progression of leukemia. It is thought that the use of autophagy inhibitors in combination can compensate for this drawback. However, drugs that promote autophagy are thought to promote the activation of autophagy beyond the maintenance of leukemia homeostasis, leading to cell death.

Autophagy plays an important role in the maintenance of LSCs and drug resistance, and it is a promising therapeutic target for myeloid leukemia (Figure 3).

However, since autophagy is essential for maintaining homeostasis in normal hematopoiesis and other tissues, there are concerns about the safety of inhibiting or activating autophagy systemically. Another issue for the future is the development of small molecule inhibitors that are highly selective for autophagy. Further analysis of the differences in the dependence on autophagy between diseases (abnormal hematopoiesis due to leukemia) and normal hematopoiesis, as well as the autophagy regulatory mechanisms specific to LSCs, will be necessary to realize truly effective leukemia treatment using autophagy.
